# An evolutionary analysis of genome expansion and pathogenicity in *Escherichia coli*

**DOI:** 10.1186/1471-2164-15-882

**Published:** 2014-10-09

**Authors:** Jon Bohlin, Ola B Brynildsrud, Camilla Sekse, Lars Snipen

**Affiliations:** Division of Epidemiology, Norwegian Institute of Public Health, Marcus Thranes gate 6, P.O. Box 4404, Oslo, 0403 Norway; Epi-Centre, Department of Food-Safety and Infection Biology, Norwegian University of Life Sciences, Ullevålsveien 72, P.O. Box 8146 Dep, Oslo, NO-0033 Norway; Norwegian Veterinary Institute, P.O.Box 750, Sentrum, N-0106 Oslo, Norway; Department of Chemistry, Biotechnology and Food Sciences, Norwegian University of Life Sciences, Ås, Norway

## Abstract

**Background:**

There are several studies describing loss of genes through reductive evolution in microbes, but how selective forces are associated with genome expansion due to horizontal gene transfer (HGT) has not received similar attention. The aim of this study was therefore to examine how selective pressures influence genome expansion in 53 fully sequenced and assembled *Escherichia coli* strains. We also explored potential connections between genome expansion and the attainment of virulence factors. This was performed using estimations of several genomic parameters such as AT content, genomic drift (measured using relative entropy), genome size and estimated HGT size, which were subsequently compared to analogous parameters computed from the core genome consisting of 1729 genes common to the 53 *E. coli* strains. Moreover, we analyzed how selective pressures (quantified using relative entropy and *dN*/*dS*), acting on the *E. coli* core genome, influenced lineage and phylogroup formation.

**Results:**

Hierarchical clustering of *dS* and *dN* estimations from the *E. coli* core genome resulted in phylogenetic trees with topologies in agreement with known *E. coli* taxonomy and phylogroups. High values of *dS*, compared to *dN*, indicate that the *E. coli* core genome has been subjected to substantial purifying selection over time; significantly more than the non-core part of the genome (*p*<*0.001*). This is further supported by a linear association between strain-wise *dS* and *dN* values (*β* = *26.94* ± *0.44*, *R*^*2*^~*0.98*, *p*<*0.001*). The non-core part of the genome was also significantly more AT-rich (*p*<*0.001*) than the core genome and *E. coli* genome size correlated with estimated HGT size (*p*<*0.001*). In addition, genome size (*p*<*0.001*), AT content (*p*<*0.001*) as well as estimated HGT size (*p*<*0.005*) were all associated with the presence of virulence factors, suggesting that pathogenicity traits in *E. coli* are largely attained through HGT. No associations were found between selective pressures operating on the *E. coli* core genome, as estimated using relative entropy, and genome size (*p*~*0.98*).

**Conclusions:**

On a larger time frame, genome expansion in *E. coli*, which is significantly associated with the acquisition of virulence factors, appears to be independent of selective forces operating on the core genome.

**Electronic supplementary material:**

The online version of this article (doi:10.1186/1471-2164-15-882) contains supplementary material, which is available to authorized users.

## Background

It has been widely documented that horizontal gene transfer (HGT) can make potentially harmless, even probiotic, bacterial species lethal [1, 2]. Considerable research has focused on how bacteria can evolve from being nonthreatening, host-independent and free-living organisms to become obligatory intracellular parasites with reduced genomes [3–9]. However, the evolutionary mechanisms explaining genome expansion due to HGT are much less documented. One reason for this is the need for a large number of fully sequenced and assembled genomes from strains of species that are particularly well suited for such analyses. The recent development of high-throughput sequencing technology has reduced sequencing costs and for many microbial species there are now multiple strains, completely sequenced and assembled, available for analyses in public databases [10]. This allowed us to explore strain-level relationships between base composition, genome size and predicted HGT in several microbial species in a recent study [11]. We found that the genome size, compared at strain-level, was predominantly correlated with genomic AT content, contrary to what has been found for prokaryotes in general [12]. Additionally, AT content correlated with predicted HGT size, which again correlated with chromosome size [11]. In this study we also analyzed the influence of selective pressures on microbial genome size using the concept of relative entropy [13, 14].

Relative entropy can be used to measure genomic distance and is computed with the Kullback–Leibler measure between observed and expected codon frequencies (see [14] for more details). The expected codon frequencies are calculated from genomic nucleotide frequencies so that decreasing distances between observed and expected codon frequencies imply increased independence between the neighboring nucleotides constituting the codons. This implies more random distributions of codon frequencies presumably due to mutations/genetic drift [13, 15]. A negative correlation between relative entropy and AT content has previously been detected in microbial genomes, implying that AT-rich genomes tend to have, on average, a more random base composition than GC-rich genomes [11, 13, 16]. The greater similarity between AT-rich genomes and random DNA sequences, with similar base compositions, is a consequence of the fact that genomic mutations are in general biased towards AT-richness [17, 18].

Horizontally transferred DNA tends to have lower relative entropy than DNA of the host chromosome. Thus, it is likely that the genomes of strains with high levels of horizontally transferred DNA will, on average, have lower relative entropy than the genomes of strains having received less HGT [13]. However, it may also suggest differences in how selective forces operate at the strain level, analogous to the general negative correlation between AT content and genome size, which appears to be largely reversed at the strain-level of bacterial species [11].

The *dN* /*dS* ratio, where *dN* describes the difference in non-synonymous substitutions between taxa and *dS* designates the difference in synonymous substitutions, has also been associated with selective pressures [19]. Indeed, a large *dS* relative to *dN* is linked to purifying selection; *dN* = *dS* is assumed to indicate neutrality of selection, while a *dN* greater than *dS* is associated with positive selection [19]. Not only does *dS* > *dN* provide an approximate quantitative measure of the selective pressures involved in purging non-synonymous substitutions resulting in reduced fitness, but the relation may also give clues about the species’ population structure [18]. Additionally, time is a central factor [19]. A recent divergence between two or more strains is often indicated by *dN* > *dS*, since such mutations are more likely to take place within a short time span [19].

It has previously been shown that purifying selection correlates with genome size for microbes above strain level [20]. In the present study we wanted to examine whether selective forces would leave a base compositional pattern in the core genomes of bacterial strains undergoing genome expansion, mediated through HGT, since such a pattern has been observed for microbial species undergoing genome reduction [3, 13]. We focused our analysis on *E. coli* since this particular species is renowned for extensive HGT and has many strains sequenced and fully assembled [21, 22]. Since pathogenicity has been linked with HGT [1, 2] we also wanted to test whether the pathogenic potential of the *E. coli* strains correlated with genomic properties such as AT content, genome size, genomic drift, and selective pressures, as estimated using relative entropy and *dN*/*dS*. To reach our aim we extracted the *E. coli* core genome, consisting of 1729 genes, from 53 *E. coli* strains and estimated *dS* and *dN*, as well as the other genomic properties mentioned above. We also generated a maximum likelihood tree based on mutations in the *mutT* gene, which has been associated with hyper-mutable strains [23], and compared the congruency of that tree to the trees resulting from the *dS*- and *dN*-based hierarchical cluster analyses.

## Results and discussion

### Estimation of *dN*and *dS*from the *E. coli*core genome

We wanted to explore whether there was a relationship between the selective pressures that the *E. coli* core genome has been subjected to and genome expansion due to HGT since an association between purifying selection and genome size has previously been identified for microbial species in general [20].

**Figure 1:**
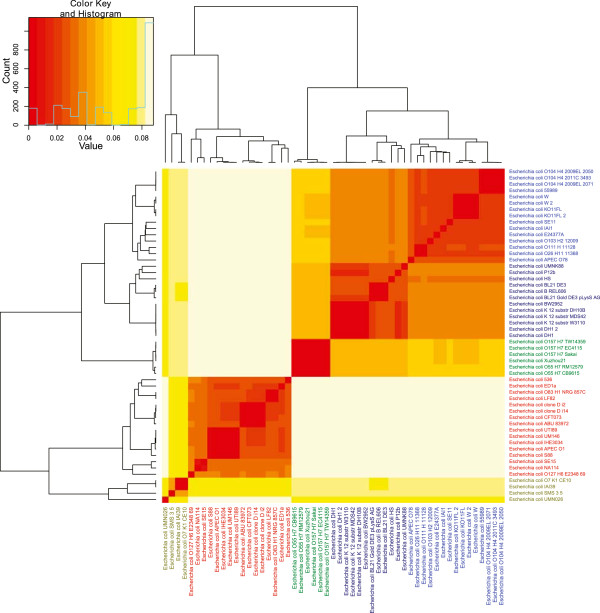
***dS*** **-based heatmap.** The heatmap demonstrates a hierarchical cluster analysis of estimated *dS* (the rate of synonymous distributions between taxa) of 1729 core genes from the 53 *E. coli* genomes. The differently colored labels designate phylogroups: D (light green), B2 (red), E (green), B1 (blue), and A (dark blue). Groups D, B2 and E consisted predominantly of pathogens; Group A was almost exclusively non-pathogenic, while Group B1 consisted of a mixture of pathogenic and non-pathogenic strains.

**Figure 2:**
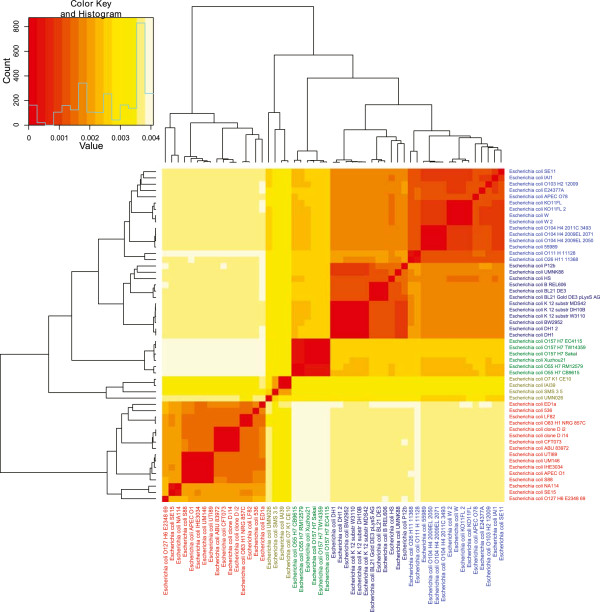
***dN*** **-based heatmap.** The heatmap shows a hierarchical cluster analysis of estimated *dN* (the rate of non-synonymous distributions between taxa) of 1729 core genes from the 53 *E. coli* genomes. The differently colored labels designate phylogroups: D (light green), B2 (red), E (green), B1 (blue), and A (dark blue). Groups D, B2 and E consisted predominantly of pathogens; Group A was almost exclusively non-pathogenic, while Group B1 consisted of a mixture of pathogenic and non-pathogenic strains.

We performed hierarchical cluster analyses based on *dS* and *dN* estimations from all 1729 genes belonging to the *E. coli* core genome. The results can be seen in Figures 1 and 2 for *dS* and *dN* estimations, respectively, and the resulting cluster groups (denoted by different colors in both Figures 1 and 2) indicate a strong association with known *E. coli* phylogroups [24–26]. Table 1 contains more information on the different *E. coli* strains and the corresponding patho-/phylo-groups resulting from the *dS* and *dN* based cluster analyses.

**Table 1:** **Information about the different** ***E. coli*** **strains used in the study**

Name	Pathogroup	Phylogroup
*Escherichia coli* O7:K1 CE10	ExPEC (neonatal meningitis)	D
*Escherichia coli* IAI39	ExPEC (Uropathogenic *E. coli* (UPEC))	D
*Escherichia coli* SMS 3-5	Multi-resistant	D
*Escherichia coli* UMN026	ExPEC (UPEC)	D
*Escherichia coli* 536	ExPEC (UPEC)	B2
*Escherichia coli* ED1A	Non-pathogenic	B2
*Escherichia coli* O83:H1 NRG 857C	AIEC (adherent-invasive E. coli)	B2
*Escherichia coli* LF82	AIEC	B2
*Escherichia coli* clone D i2	ExPEC (UPEC)	B2
*Escherichia coli* clone D i14	ExPEC (UPEC)	B2
*Escherichia coli* CFT073	ExPEC (UPEC)	B2
*Escherichia coli* ABU 83972	ExPEC (UPEC)	B2
*Escherichia coli* UTI89	ExPEC (UPEC)	B2
*Escherichia coli* UM146	AIEC	B2
*Escherichia coli* IHE3034	ExPEC (neonatal meningitis)	B2
*Escherichia coli* APEC O1	Avian pathogenic *E. coli* (APEC)	B2
*Escherichia coli* S88	ExPEC (neonatal meningitis)	B2
*Escherichia coli* SE15	Non-pathogenic	B2
*Escherichia coli* NA114	ExPEC (multidrug-resistant UPEC)	B2
*Escherichia coli* E2348_69 O127:H6	Enteropathogenic *E. coli* (EPEC)	B2
*Escherichia coli* O157:H7 TW14359	Shiga toxin-producing *E. coli* (STEC/EHEC)	E
*Escherichia coli* O157:H7 EC4115	STEC/EHEC	E
*Escherichia coli* O157:H7 Sakai	STEC/EHEC	E
*Escherichia coli* Xuzhou21	STEC/EHEC	E
*Escherichia coli* O55:H7 RM12579	Atypical EPEC (aEPEC)	E
*Escherichia coli* O55:H7 CB9615	aEPEC	E
*Escherichia coli* UMNK88	Enterotoxigenic *E. coli* (ETEC)	A
*Escherichia coli* P12b	Non-pathogenic	A
*Escherichia coli* HS	Non-pathogenic	A
*Escherichia coli* BL21 DE3	Lab strain – Non-pathogenic	A
*Escherichia coli* B REL606	Lab strain – Non-pathogenic	A
*Escherichia coli* BL21 Gold DE3 pLysS AG	Lab strain – Non-pathogenic	A
*Escherichia coli* BW2952	Lab strain – Non-pathogenic	A
*Escherichia coli* K12 substr DH10B	Lab strain – Non-pathogenic	A
*Escherichia coli* K12 substr MDS42	Lab strain – Non-pathogenic	A
*Escherichia coli* K12 substr W3110	Lab strain – Non-pathogenic	A
*Escherichia coli* DH1 (AP012030.1)	Lab strain – Non-pathogenic	A
*Escherichia coli* DH1 (CP001637.1)	Lab strain – Non-pathogenic	A
*Escherichia coli* O104:H4 str. 2009EL-2050	Enteroaggregative – EHEC (EAggEC-EHEC)	B1
*Escherichia coli* O104:H4 str. 2009EL-2071	EAggEC-EHEC	B1
*Escherichia coli* O104:H4 str. 2011C-3493	EAggEC-EHEC	B1
*Escherichia coli* 55989	EAggEC	B1
*Escherichia coli* W (CP002185.1)	Lab strain	B1
*Escherichia coli* W (CP002967.1)	Lab strain	B1
*Escherichia coli* KO11FL_162099 (CP002516.1)	Lab strain	B1
*Escherichia coli* KO11FL_162099 (CP002970.1)	Lab strain	B1
*Escherichia coli* SE11	Non pathogenic	B1
*Escherichia coli* IAI1	Non pathogenic	B1
*Escherichia coli* E24377A	ETEC	B1
*Escherichia coli* O103:H2 str. 12009	STEC/EHEC	B1
*Escherichia coli* O111:H- str. 11128	STEC/EHEC	B1
*Escherichia coli* O26:H11 str. 11368	STEC/EHEC	B1
*Escherichia coli* APEC O78	APEC	B1

From the heatmaps in Figures 1 and 2 it can be seen that *dS* is considerably higher than *dN* implying that the *E. coli* core genome has been subjected to strong purifying selection [27]. Since the core genome consists of all genes common to all the strains discussed here, these genes are presumably important for the species survival and the removal of fitness-reducing mutations appears to have been of considerable importance for the evolution of the different lineages.

**Figure 3:**
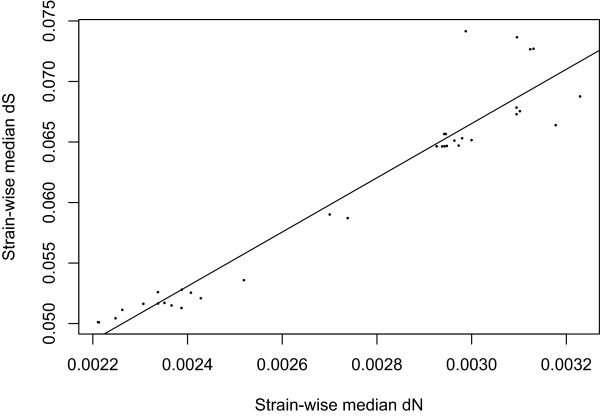
**Regression plot of strain-wise median** ***dS*** **and** ***dN.*** The figure shows median *dS* estimates plotted against median *dN* estimates for the *E. coli* strains in the study. The diagonal line designates the estimated regression line. All similar and clonal strains were removed for the regression analysis resulting in a sample size of 36 strains.

The similar topology of the heatmaps in Figures 1 and 2 points to corresponding differences in *dS* and *dN* for each strain and Figure 3 demonstrates that we found a strong linear association between median- *dS* and *dN* values with respect to each strain (*β* = *26.94* ± *0.44*, *R*^*2*^ ~ *0.98*, *p* < *0.001*). From Table 1, as well as Figures 1 and 2, it can be seen that group B1, which includes 9 pathogens and 6 non-pathogens, is closest to group A consisting almost exclusively of non-pathogens (one pathogen and 11 non-pathogens). The predominantly pathogenic groups: B2, D and E cluster together and are further away from groups A and B1. The strong correlation between *dS* and *dN* observed in Figure 3 may indicate that the formation of the different lineages may be of a more ancient origin since both *dN* and *dS* based cluster analyses resulted in cluster groups completely congruent with the established *E. coli* phylogroups [24].

**Figure 4:**
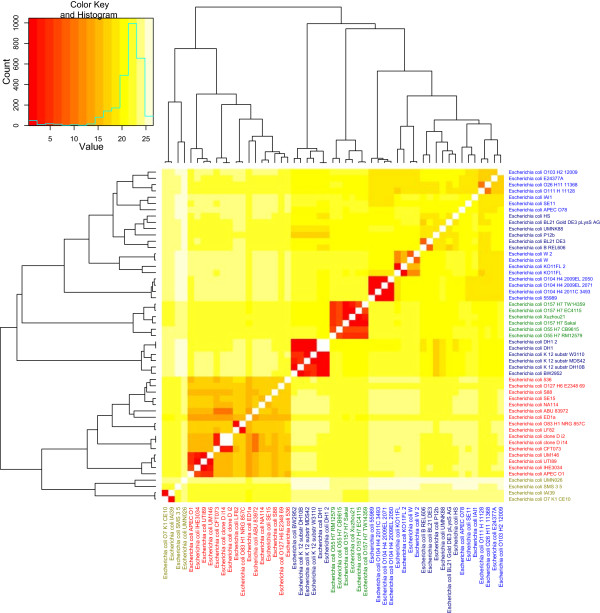
***dS*** **/** ***dN*** **-based heatmap.** The heatmap demonstrates a hierarchical cluster analysis of estimated *dS*/*dN* (the rate of synonymous to non-synonymous substitutions between taxa) of 1729 core genes from the 53 *E. coli* genomes. The differently colored labels designate phylogroups: D (light green), B2 (red), E (green), B1 (blue), and A (dark blue). Groups D, B2 and E consisted predominantly of pathogens; Group A was almost exclusively non-pathogenic, while Group B1 consisted of a mixture of pathogenic and non-pathogenic strains. The horizontal axis of the color key legend indicates multiples of *dS* to *dN*, where values close to 1 designates neutrality of selection.

As mentioned above, the type of selective pressures affecting genomes can be inferred from the *dN*/*dS* ratio. To examine the selective pressures operating on the different *E. coli* lineages we used the *dS*/*dN* ratio instead of *dN*/*dS* for clarification [19]. The resulting *dS*/*dN* heatmap can be seen in Figure 4 and shows that although phylogroups D, B2 and E cluster together, phylogroup A is divided into two cluster groups one of which is flanked by phylogroup B1. Interestingly, a similar tree topology was observed by Didelot et al. [24] in a cluster analysis based on *E. coli* non-core gene content, as opposed to the core genome *dS*/*dN* ratios explored in the present study, which may suggest that the lineages represented by phylogroups A and B1 have been exposed to similar selective pressures. Indeed, Didelot et al. points out that the frequency of HGT between these two phylogroups is higher than that observed between any of the other *E. coli* phylogroups. Therefore it is conceivable that the strains in phylogroups A and B1 are often found within geographic proximity and in similar environments [24].

### Phylogenetic inferences from the *mutT*gene

**Figure 5:**
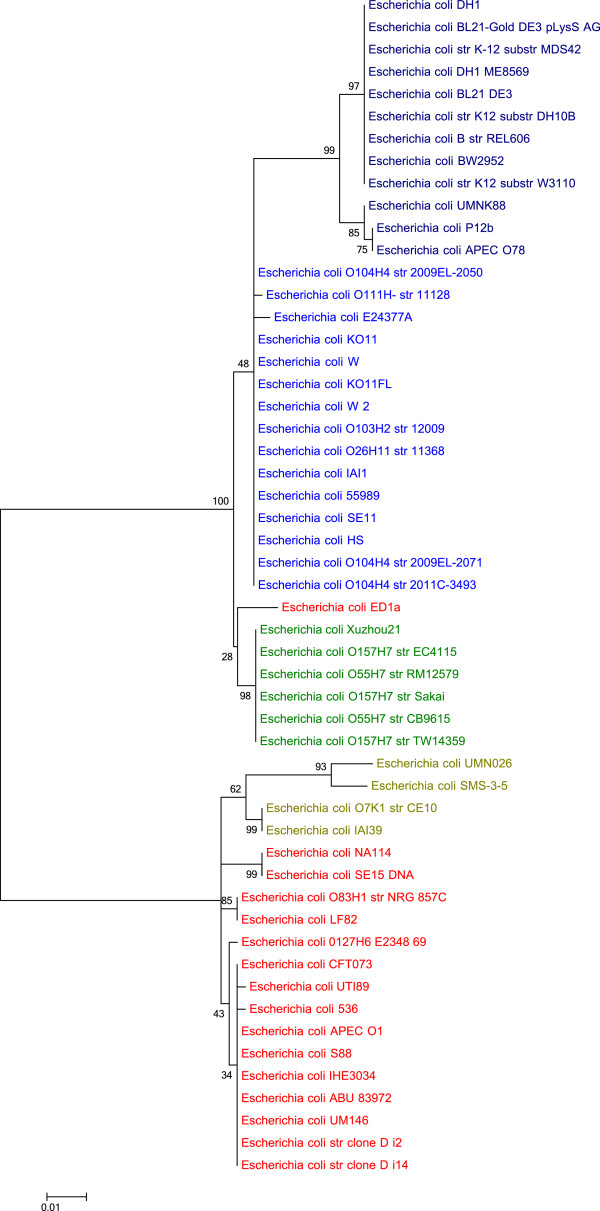
***mutT*** **based phylogenic tree.** The phylogenic tree is based on alignments of the *mutT* gene found in the core genome of all 53 *E. coli* strain. The numbers close to the branches represent bootstrap support. The differently colored labels designate phylogroups: D (light green), B2 (red), E (green), B1 (dark blue), and A (blue). Groups D, B2 and E consisted predominantly of pathogens; Group A was almost exclusively non-pathogenic, while Group B1 consisted of a mixture of pathogenic and non-pathogenic strains.

The topology of the phylogenies resulting from the *dS* and *dN* based cluster analyses (Figures 1 and 2) are congruent with the tree depicted in Figure 5, based on variants of the *mutT* gene, some of which are known to be associated with hyper-mutable *E. coli* strains [23]. The *E. coli* ED1a strain, one of two non-pathogens in phylogroup B2 (see Table 1), cluster outside all groups. Other genes related to the genomic mutation levels such as *mutY*, *mutL* and *mutM* [28] resulted in trees with topologies similar to the one we obtained with the *mutT* gene. However, these alignments were based on relatively large sequences with few mutations resulting in a bootstrap support that was too low for any consistent tree-topology to be inferred. Nevertheless, it is interesting to note that the *mutT* based-tree supported the *dN* and *dS* based phylogenies obtained above. This could mean that the different phylogroups have distinct variants of the *mutT* gene that coincide with both non-synonymous and synonymous substitution rates in the *E. coli* core genome. However, our data does not allow any conclusive statements with respect to potential effects of *mutT* genotypes on *dN*/*dS* values.

### Examination of the base composition in the *E. coli*core genome

As previously mentioned, the selective pressures that the *E. coli* core genome has been exposed to can be analyzed using relative entropy [13]. The genomic frequencies of codons subjected to strong selective pressures are assumed to be substantially different than the corresponding products of nucleotide frequencies. Conversely, codons exposed to weak selective pressures will presumably have more similar frequencies to the corresponding product of nucleotide frequencies due to mutational bias [13, 29]. The relative entropy measure cannot separate between positive- and negative selective pressures associated with *dN*/*dS*-based methods. Therefore, with regards to relative entropy, selective pressures will denote both positive- and negative selective pressures.

**Figure 6:**
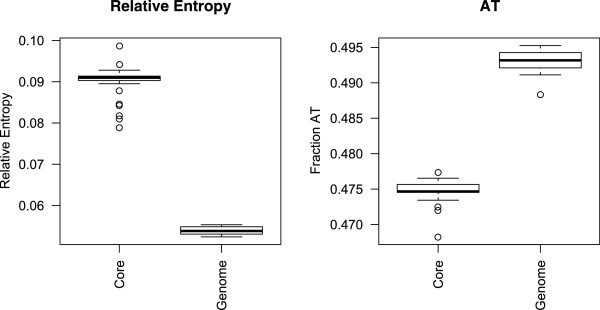
**Core genome relative entropy and AT content.** The figure consists of two panels of boxplots displaying the difference between core- and whole genome relative entropy (left), and core- and whole genome fraction of AT content (right) in all 53 *E. coli* strains.

We wanted to examine whether we could find base compositional differences between core- and whole genomes and whether properties deduced from the core-genomic base composition could be associated with corresponding whole genome properties. For the following statistical analyses we removed all strains that are known to be modified clones, or otherwise genetically very similar, to reduce bias. Details about the specific isolates included in these analyses can be found in Additional file 1. From Figure 6, left panel, it can be seen that relative entropy in the *E. coli* core genomes was significantly higher than for the corresponding whole genomes (*R*^*2*^ ~ *0.98*, *p* < *0.001*). This indicates that the core genome has been subjected to substantially stronger selective pressures than the non-core part of the genome. As previously shown (Figures 3 and 4) the *dS* estimates were substantially larger than *dN* estimates, which suggest strong selection of the purifying type. Additionally, it can be seen from Figure 6, right panel, that the core genome was significantly less AT rich (*R*^*2*^ ~ *0.98*, *p* < *0.001*) than the rest of the genome. This finding has also been linked to increased selection in other studies although of an unspecified type [30]. In the instance discussed here, relating to the *E. coli* core genome, there seems to be a connection between purifying selection and decreased AT content.

We also examined whether there was any association between core- and whole genome levels of both relative entropy and AT content, which could point towards similar selective pressures operating on the core- and whole genome. Our findings indicate no correlation between core- and whole genome relative entropy (*p* ~ *0.26*) suggesting that selective pressures operating on the core genome are most likely unrelated to selective forces effective on the rest of the genome. Core- and whole genome AT content may be negatively correlated (*p* ~ *0.058*), albeit weakly. Since this negative correlation was produced with robust regression, the result was somewhat surprising. An extra generalized additive model (GAM) [31] was therefore fitted, since such models are more capable of modeling non-linear relations, but the association between core- and whole genome AT content was no longer statistically significant (*p* ~ *0.23*). Hence, these results seem to suggest that different selective pressures form the *E. coli* core and non-core genomes.

### The effect of selective pressures on *E. coli*genome size

**Figure 7:**
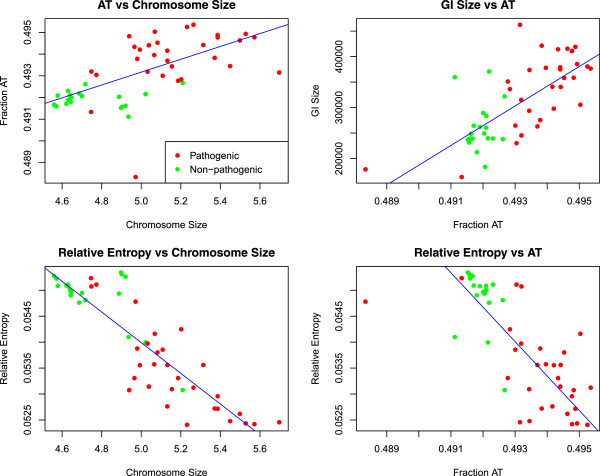
**Statistical analyses of genomic properties in 53** ***E. coli*** **strains.** The figure consists of 4 panels showing different associations between selected genomic properties of pathogenic (red dots) and non-pathogenic (green dots) *E. coli* strains. The blue line denotes the estimated regression line, which was significant for all panels (*p* < *0.05*). Top left panel shows genomic AT content versus chromosome size, while the top right panel depicts estimated HGT size versus genomic AT content. Bottom right panel designates whole genome relative entropy versus genome size, and bottom left panel shows whole genome relative entropy plotted against genomic fraction of AT.

Since we have explored how selective pressures operate on whole and core genomes using both *dN*/*dS* and relative entropy, we have the necessary results to examine whether selective forces are associated with genome size in *E. coli*. Previously, a negative correlation between *E. coli* strains and relative entropy was established which may give the impression that an increase in genome size is a consequence of reduced selective pressures. However, foreign DNA sequences incorporated into a host genome typically have lower relative entropy. Accordingly, the negative association between genome size and relative entropy may be due to the lower relative entropy of the foreign DNA [11]. In Figure 7, it can be seen that the negative association between relative entropy and genome size still holds (*R*^*2*^ = *0.72*, *p* < *0.001*). In addition, we also found a significant association between the pathogenicity status of the *E. coli* strains and genome size (*p* ~ *0.001*), AT content (*p* < *0.001*), whole genome relative entropy (*p* < *0.001*), as well as size of predicted HGT (*p* ~ *0.005*). Hence, we find a clear association between the acquisition of virulence factors and DNA uptake as well as genome size, suggesting that the pathogenic potential of *E. coli* is associated with DNA uptake. However, we could not find any association between core genome relative entropy on one hand, and genome size (*p* ~ *0.98*) or predicted GI size (*p* ~ *0.37*) on the other. Thus, the lack of correlation between core- and whole genome relative entropy in *E. coli* seems to suggest that the acquisition of foreign DNA, and virulence factors in particular, is not a consequence of increased selective pressures operating on the genome, at least not on a larger time scale. In this respect, it is interesting that *Shigella* spp., which is more or less a distinct lineage of *E. coli*, has been shown to obtain its pathogenic traits through reductive evolution and relaxed selective pressures [9]. It should also be noted that due to strong bi-modality in the strain-based *dN*/*dS* estimations no reliable statistical tests could be performed between group-wise median- *dN* and *dS* and any corresponding genomic property such as AT content, genome size and relative entropy. Plotting both *dN* and *dS* or *dN*/*dS* values against the genomic properties discussed above did not reveal any indications of potential trends and were therefore excluded.

### Genome expansion and genome reduction in *E. coli*

To our knowledge, there are no previous studies of evolutionary forces responsible for genome expansion due to HGT. A recent study discusses evolutionary aspects of recombination in recently emerged clonal *Staphylococcus aureus* and *Clostridium botulinum* isolates by examining *dN* and *dS* of SNPs in core-, non-core- and recombined DNA, but does not deal with genome expansion as such [32]. Our findings suggest that the *E. coli* core genome has been subjected to substantial selective pressures over time compared to the genome as a whole. The linear association between median *dS* and *dN* estimations for all strains indicates that purifying selection has been directing *E. coli* lineage evolution and the comparably low rates of non-synonymous substitutions (*dN*) may indicate that the core genome has remained intact for a longer time span [19]. It should also be noted that all *E. coli* strains examined in this study are publicly available whole genome sequences, and the fact that they have been selected for sequencing may be due to some special traits not commonly observed in wild-type *E. coli*.

## Conclusions

Our results support previous studies arguing that acquisition of traits through HGT may be a consequence of “spandrel”-like evolutionary processes [33] where the functions of acquired genes are formed through positive selection over time or eventually lost [34, 35]. Hence, increase of selective pressures appears not to be the driving force behind chromosome expansion and acquisition of new traits in *E. coli*, which is consistent with related findings, also those pointing to an analogous evolutionary trail for gene duplications [36, 37]. Pathogenic *E. coli* may thus have evolved as a consequence of a hostile environment, where virulence associated genes are abundant. We anticipate that a lot more will be said about this in the future.

## Methods

59 *E. coli* genomes, with their annotated coding genes and corresponding proteins, were downloaded from NCBI/Genbank [10]. In six of the genomes we discovered a lack of correspondence between the coding genes and their listed proteins, and these six genomes were discarded from the downstream analysis. See Additional file 1 for more information on the different *E. coli* strains used in the study. Genomic properties such as genome size, AT content and relative entropy were estimated using in-house scripts that are available upon request. All statistical analyses were performed with R [38].

### Extraction of the core genome

All proteins from every genome were BLASTed (blastp) [39] against all proteins of all other genomes, and a distance was computed between all protein pairs as described in [40]. Based on these distances, proteins were clustered using hierarchical clustering with complete linkage, and divided into clusters by cutting the dendrogram tree at distance 0,1. Loosely speaking, this means any two proteins in the same cluster share 90% similarity. Using this rather strict cutoff resulted in a set of 1729 core clusters, *i.e*. clusters with at least one protein for each of the 53 genomes in the study. Next, paralogs were eliminated from each cluster using the same procedure as described in [40]. The 53 orthologs in each cluster were aligned using the MCoffee software [41] and the protein-alignments were back-translated to DNA-alignments using the TranslatorX software [42].

### Estimation of core genome *dN*and *dS*

To calculate *dN* and *dS* we followed the method first described by Li et al. [43]. Briefly, we sequentially performed gene-wise multiple alignments as described above on all 1729 core genes from the 53 strains used in the study. The alignment ends were trimmed manually so that the sequences within the alignments were all of the same length. We then used the *seqinr* package [44] in R to read the alignments, and subsequently calculated gene-by-strain *dN* and *dS* values using the *kaks*() command. For strain-wide assessments, the *dN* and *dS* estimates for individual genes were added up and weighted according to gene length. *dN* and *dS* for each strain were based on the median from all versus all comparisons.

Due to the bimodal distribution of the core genome-based *dN* and *dS* values, heatmaps based on hierarchical clustering with Euclidean distance were created for each of the *dS*, *dN* and *dS*/*dN* estimated distance matrices so that potential differences between the strains could be examined. These matrices are included in an R-file (see Additional file 2).

### Relative entropy

Relative entropy measures the Kullback–Leibler divergence between observed and expected codon frequencies in coding regions [13], *i.e*.:


where the sum is taken over all 64 possible codons *XYZ* consisting of nucleotides *X*, *Y* and *Z*, respectively. *F*_*i*_ is a function returning the frequency of codon *XYZ*, or nucleotides *X*, *Y* and *Z*, from genome *i*. A low *D*_*KL*_ indicates that the observed codon frequencies are, on average, similar to the individual nucleotide frequencies, signifying that the codon frequencies are more random, presumably due to relaxation of the selective forces operating on the genome [13].

### The *mutT*based phylogenetic tree

The phylogenic tree based on the *mutT* gene was created, after sequence alignment, using maximum likelihood estimation and 500 bootstraps using the package Mega 6 [45]. Based on statistical analyses carried out with the “Ape” package in R [46], we found that a nucleotide substitution model based on the Tamura-Nei 93 model [47], which assumes equal transversion rates and unequal transition rates, with Gamma-distributed among-site rate variation was the model with the lowest AIC [48] and therefore chosen. The Gamma distribution was discretized into 6 categories, which is the default number of categories; changes to this number did not notably affect the tree topology. The DNA sequences which the *mutT* based phylogenetic tree is based on are included in FASTA-format (Additional file 3).

### HGT predictions

HGT predictions based on the SIGI-HMM method were downloaded and computed using the *Islandviewer* webpage [49–51].

### Statistical analyses

The statistical analyses were carried out using an iterative robust MM-type regression (M-type estimator with Tukey’s biweight and initial coefficient estimates provided from an S-type estimator) [52] with significance estimates (*p*-values) obtained from *t*-statistics. All similar strains were discarded before these statistical analyses so that the sample size was reduced to 36 strains (see Additional file 4 and Additional file 5). Robust regression was used where there were several outlying residuals resulting in moderately skewed distributions, otherwise standard ordinary least squares regression was used, which additionally includes a goodness-of-fit estimate (*R*^*2*^). The association between core- and whole genome AT content was also tested using a generalized additive model (GAM), where the predictor (whole genome AT content) was modeled using a spline-function [53]. Additional file 5 contains all estimates resulting from the statistical analyses.

## Electronic supplementary material

Additional file 1: **An Excel file containing information about the strains used in the study.** (XLSX 15 KB)

Additional file 2: **An R-file with the dN/dS estimates used in the cluster analyses.** (ZIP 30 KB)

Additional file 3: **FASTA file with aligned sequences used to make mutT-based tree.** (ZIP 1 KB)

Additional file 4: **An Excel file with data used for regression analyses.** (XLSX 18 KB)

Additional file 5: **An Excel file containing data from the regression analyses.** (XLSX 11 KB)
